# The molecular mechanism on suppression of climacteric fruit ripening with postharvest wax coating treatment *via* transcriptome

**DOI:** 10.3389/fpls.2022.978013

**Published:** 2022-08-15

**Authors:** Yajing Si, Tianxing Lv, Hongjian Li, Jiaojiao Liu, Jiamao Sun, Zhaohui Mu, Junling Qiao, Haidong Bu, Hui Yuan, Aide Wang

**Affiliations:** ^1^Key Laboratory of Fruit Postharvest Biology (Liaoning Province), Key Laboratory of Protected Horticulture (Ministry of Education), National and Local Joint Engineering Research Center of Northern Horticultural Facilities Design and Application Technology (Liaoning), College of Horticulture, Shenyang Agricultural University, Shenyang, China; ^2^Liaoning Institute of Pomology, Xiongyue, China; ^3^Mudanjiang Branch, Heilongjiang Academy of Agricultural Sciences, Mudanjiang, China

**Keywords:** *Malus domestica*, *Pyrus ussuriensis*, fruit wax coating, fruit ripening, RNA-seq

## Abstract

Wax coating is an important means to maintain fruit quality and extend fruit shelf life, especially for climacteric fruits, such as apples (*Malus domestica*). Here, we found that wax coating could inhibit ethylene production, chlorophyll degradation, and carotenoid synthesis, but the molecular mechanism remains unclear. The regulatory mechanism of wax coating on apple fruit ripening was determined by subjecting wax-treated apple fruits to transcriptome analysis. RNA-seq revealed that 1,137 and 1,398 genes were upregulated and downregulated, respectively. These differentially expressed genes (DEGs) were shown to be related to plant hormones, such as ethylene, auxin, abscisic acid, and gibberellin, as well as genes involved in chlorophyll degradation and carotenoid biosynthesis. Moreover, we found that some genes related to the wax synthesis process also showed differential expression after the wax coating treatment. Among the DEGs obtained from RNA-seq analysis, 15 were validated by quantitative RT-PCR, confirming the results from RNA-seq analysis. RNA-seq and qRT-PCR of pear (*Pyrus ussuriensis*) showed similar changes after wax treatment. Our data suggest that wax coating treatment inhibits fruit ripening through ethylene synthesis and signal transduction, chlorophyll metabolism, and carotenoid synthesis pathways and that waxing inhibits endogenous wax production. These results provide new insights into the inhibition of fruit ripening by wax coating.

## Introduction

Fruit ripening is a very important physiological process during fruit development and is often accompanied by physiological changes in fruit color, firmness, odor, and sugar-acid ratio (Lelièvre et al., 1997). These changes are affected by environmental factors, gene regulation, and biotic and abiotic stresses (Klee and Giovannoni, 2011; Larrainzar et al., 2014). Some fruits produce a large amount of ethylene during ripening and are called climacteric fruits, while others produce little ethylene and are called non-climacteric fruits (Adams-Phillips et al., 2004; Farcuh et al., 2018). The climacteric fruit undergoes a climacteric change and a peak of ethylene release during storage, leading to faster fruit decay and shortening the storage period (Kou and Wu, 2018).

The storage period of fruits is associated with the level of ethylene released during the ripening process (Osorio et al., 2013; Farneti et al., 2022). Reducing ethylene production delays fruit ripening and prolongs shelf life. 1-aminocyclopropane-1-carboxylic acid synthase (*ACS*) and 1-aminocyclopropane-1-carboxylic acid oxidase (*ACO*) genes are the key genes involved in ethylene synthesis. In the pear (*Pyrus ussuriensis*) fruit, ethylene treatment can induce four *ACS* genes and three *ACO* genes (Yuan et al., 2020). *MdACS1*-silenced transgenic apple (*Malus domestica*) fruits had little ethylene production during ripening and significantly prolonged storage (Dandekar et al., 2004). In addition to ethylene, many hormones are also involved in fruit ripening. The Gretchen GH3 (GH3) protein controls auxin homeostasis, and silencing of *AcGH3.1* delays fruit softening during postharvest kiwifruit (*Actinidia chinensis*) (Gan et al., 2019). Auxin application before the commercial harvest stage induces apple (*Malus domestica*) fruit ripening (Yue et al., 2020). Abscisic acid (ABA) is a ripening regulator in non-climacteric fruits and is associated with ripening in climacteric fruits. In the apricot (*Prunus armeniaca* L.) fruit, exogenous ABA treatment can significantly induce the expression of the ABA receptor gene *PaPYL9*, and the overexpression of *PaPYL9* in tomato fruit can promote early ripening (Jia et al., 2021). It has been reported that endogenous gibberellin content decreases during tomato (*Solanum lycopersicum*) fruit ripening, and the application of gibberellin (GA) biosynthesis inhibitors during the green ripening stage of fruit can accelerate fruit ripening (Li H. et al., 2019). Some researchers also applied GA combined with cytokinin to pineapple (*Ananas comosus*) fruits after flowering induction treatment and found that they could improve fruit quality and delay fruit ripening (Suwandi et al., 2016).

Chlorophyll I (ChI) degradation and carotenoid synthesis are common natural phenomena during fruit ripening. Ethylene can activate E3 ubiquitin ligase *MdPUB24* in the apple fruit to interact with and ubiquitinate *MdBEL7*, resulting in chlorophyll degradation during fruit storage (Wei et al., 2021). Exogenous ethylene treatment can strongly promote the expression of carotenoid biosynthesis genes in tomatoes (Cruz et al., 2018). Therefore, chlorophyll degradation and carotenoid synthesis are thought to be regulated by maturation.

Wax is a layer of wax film formed on the surface of the fruit, and is closely related to the quality and storage of the fruit as a natural protective layer. Removing the epidermal wax of postharvest blueberry fruit accelerates water loss and fruit rot and shortens the storage period (Chu et al., 2018). In tomato fruit, the key gene for wax synthesis, *CER1-1*, catalyzes the biosynthesis of waxy alkanes, which can improve the shelf life and drought tolerance of the fruit (Wu et al., 2022). In zucchini (*Cucurbitaceae Trichosanthes* L.) fruits, the alkane biosynthesis pathway and epidermal morphology play an important role in the postharvest fruit quality (Carvajal et al., 2021).

Artificial fruit wax is a preservative used to improve fruit quality. It is mainly made of animal and vegetable wax as film-forming agents or emulsified with animal and vegetable wax. Postharvest wax coating is an effective method to avoid the loss of fruit quality. Waxing is convenient to operate and easy to popularize, which cannot only improve fruit quality but also prolong fruit storage period and shelf life. Postharvest waxing of pineapple (*Ananas comosus*) can delay fruit color change, effectively improve fruit quality, and inhibit fruit ripening (Li X. et al., 2018). Wax coating treatment of jujube fruit (*Ziziphus jujuba* Mill) and passion fruit (*Passiflora edulis* Sims) also produced similar results (Rashwan et al., 2020; Sang, 2020). Researchers have found that wax coating together with low-temperature storage better prolongs the shelf life of cucumber (*Cucumis sativus*) fruit (Li J. et al., 2018). In mango (*Mangifera indica*) fruit, waxing combined with nitric oxide (NO) can maintain fruit firmness at 13°C for 18 days (Vázquez-Celestino et al., 2016).

These studies suggest that postharvest wax coating treatment can inhibit the ripening of climacteric fruits; however, corresponding changes in the gene expression and their molecular mechanisms are poorly understood. Hence, we aimed to undertake an in-depth analysis of waxed and unwaxed apple and pear fruits using transcriptome sequencing technology to gain new insights into the molecular basis of the inhibition of climacteric fruit ripening by wax coating.

## Materials and methods

### Plant materials and treatments

Apple (*Malus domestica* cv. Golden Delicious [GD]) fruits were harvested from the Experimental Orchard of Liaoning Pomology Institute (Xiongyue, China). Mature, pest-free, and uniform-sized GD fruits were collected on commercial harvest day (145 days after flowering, DAFB) and immediately transported to the laboratory. The apples were washed with clean water and divided into two groups. The first group was treated with liquid wax that was applied evenly at a concentration of 80% to the peel and stem of the apple several times. The fruits were then dried naturally to form an even surface coating of the wax. The second group of apples remained untreated and was used as the control. The fruits in the control and treated groups were kept at 25°C for 25 days and sampled every 5 days. On each sampling day, five apples were chosen randomly from each group as biological replicates. The peel and pulp tissues of the fruit were separated, snap-frozen in liquid nitrogen, and stored at −80°C until further use.

Pear (*Pyrus ussuriensis* cv. Nanguo) fruits were harvested from an orchard farm in Anshan, Liaoning, China. After harvesting the fruits on commercial harvest day (135 DAFB), wax was applied as described above. Similar to the apple fruit, the pear fruits of the control and treatment groups were stored at 25°C for 25 days and sampled every 5 days. Since the fruit softened and could not be sampled at 25 days, the samples were taken to 20 days. On each sampling day, five pears were randomly selected from each group as biological replicates.

The liquid wax selected in this study was morpholine fatty acid salt fruit wax produced by the Beijing Institute of Chemical Industry (Beijing, China). Its main chemical components are natural palm wax (approximately 10–20%), morpholine fatty acid salt (2.5–3%), and water (85–87%). The preliminary studies found a concentration of 80% to be the optimal wax concentration for fruit treatment. Fruit wax (80%) was prepared by mixing 80 mL of morpholine fatty acid salt fruit wax with 20 mL of distilled water.

### Measurement and analysis of physiological data

#### Ethylene production rate

Ethylene measurements were taken every 5 days. The ethylene production rate of the fruits was measured as previously described (Yuan et al., 2017). Five fruits per sample were used for measurement and analysis.

#### Flesh firmness

Fruit flesh firmness measurements were taken every 5 d. It was measured with a portable pressure tester (FT-327; Facchini, Italy), and the firmness of apple and pear fruits was measured with 11 and 8 mm probes, respectively. Four pericarps (approximately 2 cm in diameter) were removed from the opposite side of each fruit. The probe was then inserted into the tissue of the cut surface to the depth of the probe graduation mark vertically and quickly. At this point, the value on the dial was read and recorded. Five fruits per sample were used for measurement and analysis.

#### Chlorophyll and carotenoid contents

A total of 80% acetone and 95% ethanol were used in a 1:1 ratio to prepare an extraction solution. Peeled samples stored at −80°C were ground into a powder. The powdered sample (0.4 g) was weighed into a 10 mL-centrifuge tube, 8 mL of the extraction solution was added, and the mixture was vortexed. Experiments were performed in triplicate. After leaching at 25°C for 24 h in the dark, the mixture was centrifuged at 8,000 × g or 15 min at 25°C, and the supernatant was collected. A spectrophotometer was used to detect the absorbance of the supernatant at 470, 649, and 665 nm (zero-adjustment was done using the extraction solution), and the following formulae were used to calculate the total chlorophyll content and carotenoid content:


(1) Chlorophylla(Ca)=13.95A-6656.8A,649

(2) Chlorophyllb(Cb)=24.96A-6497.32A,665

(3) Totalchlorophyll⁢(Ct)=Ca+Cb,

(4) $$\mathrm{Carotenoids}\,\left(\mathrm{Cx}\right)=\frac{1000\,A_{470}-2.05\,\mathrm{Ca}-114.8\,\mathrm{Cb}}{248},$$

(5) $$\mathrm{Pigmentcontent}\,\left(\mathrm{mg}/g\right)=\frac{\text{Ct}\,\left(\text{Cx}\right)\times \text{V}\times \text{N}}{\text{W}\times 1000},$$

where C is the pigment concentration (mg/L), A is the absorbance, V is the extract volume (mL), N is the dilution factor, and W is the sample weight (g).

### RNA extraction, library preparation, and RNA sequencing

RNA-seq analysis was performed on the fruit mixture of peel and pulp stored for 10 d. The first analysis was performed in 2020 and the other two in 2021, forming three biological replicates of apple fruit. Pear fruit for RNA-seq analysis was performed in 2020.

Total RNA was isolated and purified using TRIzol reagent (Invitrogen, Carlsbad, CA, United States) following the manufacturer’s procedure. The RNA amount and purity of each sample was quantified using NanoDrop ND-1000 (NanoDrop, Wilmington, DE, United States). The RNA integrity was assessed by Bioanalyzer 2100 (Agilent, CA, United States) with RIN number > 7.0, and confirmed by electrophoresis with denaturing agarose gel. Poly (A) mRNA purified from 2 μg total RNA using Dynabeads Oligo attached magnetic beads. The mRNA is fragmented into small pieces using divalent magnesium ions under elevated temperatures. Then the cleaved RNA fragments were reverse-transcribed to create the final cDNA library according to the protocol (KC-Digital™ Stranded mRNA Library Prep Kit for Illumina^®^). At last, we performed the 2 × 150 bp paired-end sequencing (PE150) on an Illumina NovaSeq™ 6000 (Wuhan Seqhealth Co., Ltd., China) following the vendor’s recommended protocol.

### Bioinformatics analysis

Raw sequencing data were transformed into valid reads after data processing. The HISAT2 software was used to map the reads to the reference apple genome^1^ and pear genome.^2^ StringTie was used to perform expression level for mRNAs by calculating FPKM in 2020, and FeatureCounts was utilized to calculate RPKMs in 2021. Genes differentially expressed between control and treated were identified using the edgeR package. The differentially expressed transcripts were selected based on log2 (fold change) > 1 or log2 (fold change) < −1 and with statistical significance (*p*-value < 0.05). The pathway analyses of GO and KEGG enrichment of DEGs were based on the Gene Ontology Database^3^ and KEGG pathway,^4^ respectively.

### qRT-PCR analysis

Total RNA was extracted according to the method of previously described (Li et al., 2014). Total RNA (800 ng) was used to synthesize first-strand cDNA using the PrimeScript First Strand cDNA Synthesis kit (TAKARA, Japan). qRT-PCR was performed as previously described (Tan et al., 2013). Specific primers for each gene were designed using Primer3.^5^ The primers used in this study are listed in Supplementary Table 1. The apple actin gene (*MdActin*) was used as the internal control. Experiments were performed in triplicate.

### Statistical analysis

The data of qRT-PCR and physiological assays were analyzed using Microsoft excel, and the results are shown as mean ± SE. Statistical significance among the means were determined with the Student’s *t*-test, and *p < 0.05, ^**^p < 0.01. All figures were prepared by Origin2018.

## Results

### Postharvest wax coating treatment inhibits the ripening of apple fruit

**FIGURE 1:**
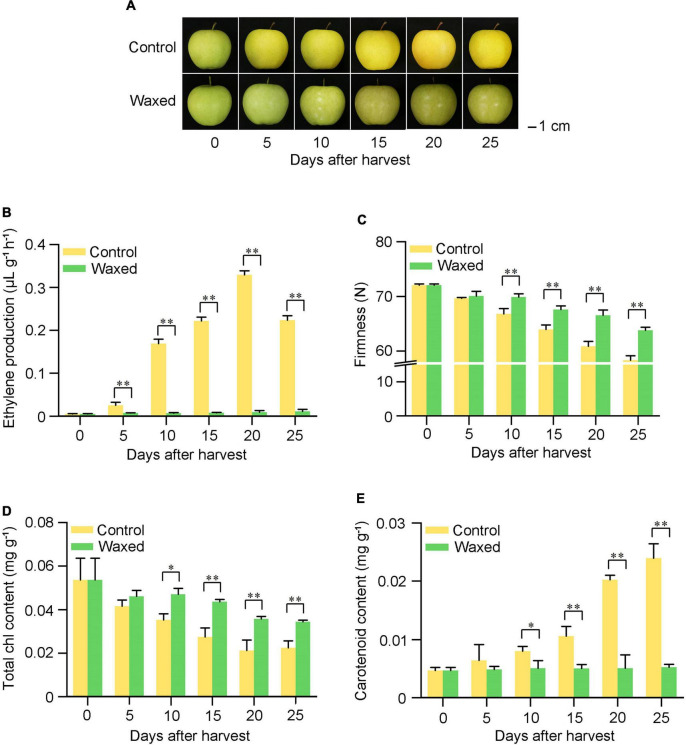
Postharvest wax coating treatment inhibits apple fruit ripening. Apple fruit were collected on the commercial harvest day (145 DAFB) in 2020 and storage at room temperature for 25 days **(A)**. After treatment, ethylene production **(B)**, fruit firmness **(C)**, total Chl content **(D)**, and carotenoid content **(E)** were measured. Control, apple fruit not receiving treatment; Waxed, fruit treated with morpholine fatty acid salt fruit wax. Scale bar = 1 cm. The x-axis represents days of storage at room temperature after harvest. Five bioligical replicates were analyzed for ethylene production, fruit firmness, and three bioligical replicates for total Chl content and carotenoid content. Values represent mean ± SE. Statistical significance was determined using a Student’s *t*-test, and *p < 0.05, ^**^p < 0.01.

After harvested on commercial day, the apple fruits were treated with wax and stored at 25°C for 25 d (Figure 1A). The untreated apple fruits showed a climacteric peak when stored for 20 d, the ethylene release reached 0.31 μL⋅g^–1^⋅h^–1^, and the waxed fruit was about 11% of that of the control (Figure 1B). The hardness of the fruit tends to decrease with ripening. During the storage period, the wax coating treatment better maintained the hardness of the fruit, and its decline was reduced (Figure 1C). Wax coating delayed the yellowing of apple fruit skin (Figure 1A). To demonstrate this phenomenon more clearly, we measured the chlorophyll and carotenoid contents of the fruit peel and found that the decrease in chlorophyll content and the increase in carotenoid content were suppressed in the wax-treated fruits (Figures 1D,E). In 2021, fruits harvested at the commercial harvest stage were also treated with liquid wax, and we observed the same effect of ripening inhibition in 2021 as in 2020 (Supplementary Figure 1).

### RNA-seq and analysis of differentially expressed genes in apple fruit

The waxed fruits showed differences in several indicators of ripeness compared to the untreated ones by day 10. To characterize DEGs associated with ripening under control and wax treatments, RNA-seq analysis was performed. A total of 36.58–50.24 million valid reads were obtained from the six samples (Control-1, Control-2, Control-3, Waxed-1, Waxed-2, and Waxed-3). A statistical summary of the three biological replicates is shown in Table 1. Six samples were prepared and sequenced as six independent RNA libraries, and 49,628,898, 57,559,864, 78,224,472, 48,782,016, 76,571,408, and 64,401,010 raw reads were obtained, respectively. The unreliable raw read data, including low-quality bases, sequencing linkers, and non-carrier errors, were excluded for obtaining high-quality, clear, and valid read data. The percentages of Q20, Q30, and GC content were more than 99.9, 98.2 and 47%, respectively. Furthermore, we used Hisat software to align the valid reads to the reference genome and found that the value of mapped reads was 93.15–97.04%, and the value of uniquely mapped reads was 81.31–97.51%. In all samples, according to the region information of the reference genome, the percentage of sequenced sequences located in exonic regions was the highest (Supplementary Figure 2A).

**TABLE 1:** Summary of RNA-sequencing of apple fruit waxing treatment.

Sample	Control-1	Control-2	Control-3	Waxed-1	Waxed-2	Waxed-3
Raw reads	49,628,898	57,559,864	78,224,472	48,782,016	76,571,408	64,401,010
Valid reads	49,141,912	36,576,146	50,244,818	48,306,536	48,207,626	40,027,470
Q20 (%)	99.99	99.95	99.95	99.99	99.95	99.95
Q30 (%)	98.23	98.35	98.35	98.28	98.35	98.35
GC (%)	47	48	48	47	48	48
Total mapped reads (%)	46,139,503 (93.89%)	34,930,093 (95.50%)	47,999,689 (95.53%)	44,997,639 (93.15%)	46,644,607 (96.76%)	38,841,043 (97.04%)
Unique Mapped reads (%)	37,697,492 (81.71%)	34,014,756 (97.38%)	46,806,107 (97.51%)	36,586,784 (81.31%)	45,365,691 (97.26%)	37,787,652 (97.29%)
Multi Mapped reads (%)	8,442,011 (18.29%)	915,337 (2.62%)	1,193,582 (2.49%)	8,410,855 (18.69%)	1,278,916 (2.74%)	1,053,391 (2.71%)

Sample, sample name; Raw reads, the total number of reads of the offline data; Valid resds, the number of reads after removal of low-quality bases, sequencing adapters, and non-carrier errors; Q20, Percentage of bases with quality value ≥ 20; Q30, Percentage of bases with quality value ≥ 30; GC, The sum of base G and C accounts for the percentage of total base number; Total mapped read: the number of reads that can be aligned to the reference genome; Unique Mapped reads, the number of reads that can only be uniquely aligned to one position in the genome; Multi Mapped reads, the number of reads that can be aligned to multiple positions in the genome.

A total of 3,744 and 6,777 DEGs were identified between the control and wax-treated groups in the RNA-seq analysis performed in 2020 and 2021, respectively (Supplementary Sheet 1). To identify specific genes that were associated with waxing, we identified the genes that were commonly upregulated and downregulated among the three biological replicates of the RNA-seq experiments performed in 2020–21 (Supplementary Sheet 2). The Venn diagrams showing these common gene numbers are presented in Supplementary Figures 2B,C.

Gene Ontology (Go) analysis was applied to analyze biological processes and functions enriched in common DEGs. The DEGs were classified into three GO categories: biological process, cellular component and molecular function. Among them, biological processes involve the most components, followed by molecular functions, and cellular components involve the least components. Among the up-regulated differential genes, nucleic acid binding, heterocyclic compound binding and organic cyclic compound binding were more abundant in molecular functions. However, among the down-regulated differential genes, lipid metabolic process, carboxylic acid metabolic process, oxoacid metabolic process and organic acid metabolic process were more abundant in biological processes (Supplementary Figure 3).

### Analysis of plant hormone-related genes in apple fruit

Compared with the control, wax-coated apples had DEGs that showed the enrichment for hormone synthesis and signal transduction processes of auxin, abscisic acid, cytokinin, gibberellin, and ethylene (Supplementary Sheet 3).

**FIGURE 2:**
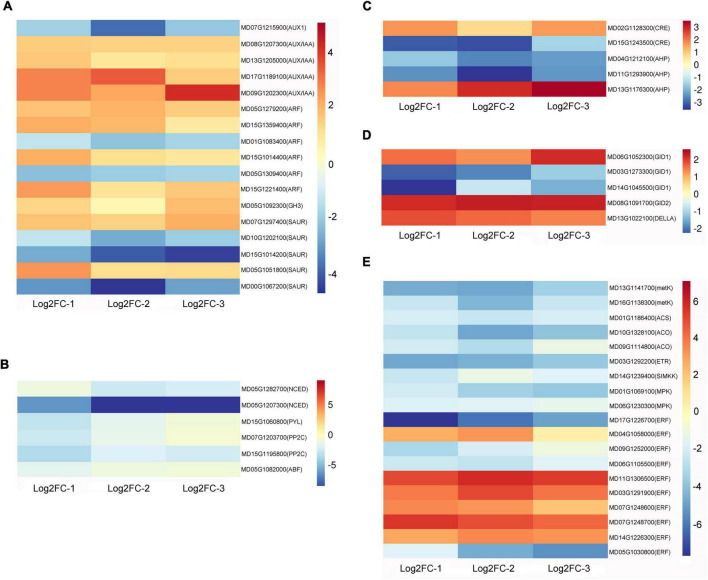
Heat maps of plant hormone-related DEGs after waxing treatment in apple fruit. Selected DEGs related to auxin **(A)**, abscisic acid **(B)**, cytokinin **(C)**, gibberellin **(D)**, and ethylene **(E)** from RNA-Seq date. Log2FC-1, Log2 (Fold Change) value of Waxed-1 and Control-1 in apple fruit RNA-Seq; Log2FC-2, Log2 (Fold Change) value of Waxed-2 and Control-2 in apple fruit RNA-Seq; Log2FC-3, Log2 (Fold Change) value of Waxed-3 and Control-3 in apple fruit RNA-Seq. The scale bar indicates upregulated (red) and downregulated (blue) DEGs.

The auxin influx carrier *AUX1* gene of the AUX1/LAX family was downregulated after fruit waxing. The auxin-responsive proteins IAA AUX/IAA showed an overall upregulated expression where four genes were found to be upregulated, and two were downregulated among auxin response factors *ARFs*. Moreover, the expression of *GH3.1* from the auxin-responsive *GH3* gene family was upregulated in wax-coated fruit, and some genes of the SAUR family proteins showed differential expression. This indicates that genes of the same family could have different functions in the context of fruit ripening (Figure 2A).

Transcriptome analysis showed that *NCED1 and NCED5* genes were downregulated after wax treatment. We found that some genes involved in ABA signaling were downregulated after treatment. These include the ABA receptor protein *PYL2*, two protein phosphatases *PP2C*, and an ABA response element-binding factor *ABF* (Figure 2B).

In the cytokinin signal transduction pathway, two CTK receptor *CRE* genes and three histidine-containing phosphotransfer protein *AHP* genes were differentially expressed following wax coating (Figure 2C).

The gibberellin receptor *GID1*, F-box protein *GID2*, and *DELLA* genes are components of the gibberellin signal transduction pathway. After waxing treatment, *GID2* and *DELLA* were upregulated; two of the three *GID1* genes were downregulated, while the other was upregulated (Figure 2D).

Ethylene is the most important hormone in the regulation of fruit ripening. In our study, 19 DEGs related to ethylene synthesis and signal transduction were identified, and a heatmap showing the differential expression of these genes is presented in Figure 2E. After wax coating, some rate-limiting enzyme genes associated with the ethylene biosynthesis pathway were identified, such as S-adenosylmethionine synthase (*metK*), 1-aminocyclopropane-1-carboxylate synthase (*ACS*), and 1-aminocyclopropane-1-carboxylate oxidase (*ACO*). This was consistent with the reduction in ethylene production after wax treatment. The ethylene receptor gene *ETR* was downregulated, and the expression of ethylene-responsive transcription factor *ERFs* showed different trends. This result indicates that wax treatment could affect the expression of ethylene-related genes and inhibit fruit ripening.

### Analysis of chlorophyll and carotenoid-related genes in apple fruit

**FIGURE 3:**
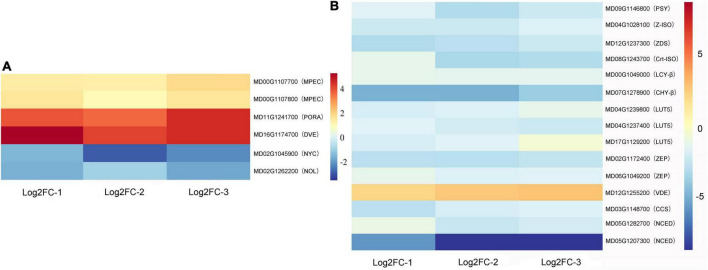
Heat maps of chlorophyll and carotenoid-related DEGs after waxing treatment in apple fruit. Selected DEGs related to chlorophyll **(A)** and carotenoid **(B)** from RNA-Seq date. Log2FC-1, Log2 (Fold Change) value of Waxed-1 and Control-1 in apple fruit RNA-Seq; Log2FC-2, Log2 (Fold Change) value of Waxed-2 and Control-2 in apple fruit RNA-Seq; Log2FC-3, Log2 (Fold Change) value of Waxed-3 and Control-3 in apple fruit RNA-Seq. The scale bar indicates upregulated (red) and downregulated (blue) DEGs.

Chlorophyll and carotenoid-related differentially expressed genes are listed in Supplementary Sheet 3. After waxing, the chlorophyll synthesis-related genes magnesium-protoporphyrin IX monomethyl ester [oxidative] cyclase (*MPEC)*, protochlorophyllide reductase (*PORA)*, and divinyl chlorophyllide a 8-vinyl-reductase (*DVR)* were upregulated, and the decomposition genes probable chlorophyll(ide) b reductase (*NYC)* and chlorophyll(ide) b reductase (*NOL)* were downregulated (Figure 3A). This provides an explanation for the physiological phenomenon of maintenance of green color in wax-coated fruits. In the heat map, we can observe that, except for the violaxanthin de-epoxidase gene *VDE*, other differential genes were downregulated in wax-coated fruits (Figure 3B). These are structural genes encoding metabolic enzymes in the carotenoid pathway, including phytoene synthase (*PSY*), ζ-carotene isomerase (*Z-ISO*), zeta-carotene desaturase (*ZDS*), carotenoid isomerase (*Crt-ISO*), lycopene-β-cyclase (*LCY*-β), beta-carotene hydroxylase (*CHY*-β), carotenoid beta-ring hydroxylase (*LUT5*), zeaxanthin epoxidase (*ZEP*), capsanthin/capsorubin synthase (*CCS*), and 9-cis-epoxycarotenoid dioxygenase (*NCED*). They are responsible for the synthesis of many types of carotenoids, such as phytoene, zeta-carotene, lycopene, alpha-carotene, lutein, gamma-carotenoids, beta-carotene, zeaxanthin, and neoxanthin. This shows that the inhibition of fruit yellowing by wax coating is related to the reduction of carotenoid content and inhibition of related synthetic genes.

### Analysis of fatty acid and wax-related genes in apple fruit

**FIGURE 4:**
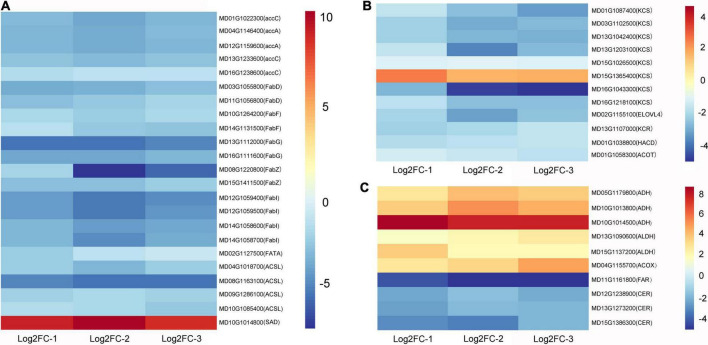
Heat maps of fatty acid and wax-related DEGs after waxing treatment in apple fruit. Selected DEGs related to fatty acid biosynthesis **(A)**, fatty acid elongation **(B)**, fatty acid degradation and wax biosynthesis **(C)** from RNA-Seq date. Log2FC-1, Log2 (Fold Change) value of Waxed-1 and Control-1 in apple fruit RNA-Seq; Log2FC-2, Log2 (Fold Change) value of Waxed-2 and Control-2 in apple fruit RNA-Seq; Log2FC-3, Log2 (Fold Change) value of Waxed-3 and Control-3 in apple fruit RNA-Seq. The scale bar indicates upregulated (red) and downregulated (blue) DEGs.

The expression trends of some genes in the fatty acid synthesis, elongation, degradation, and wax synthesis pathways were altered after wax coating (Supplementary Sheet 3). Based on the enrichment of genes in the KEGG database, genes involved in the fatty acid synthesis pathway, such as carboxylase *accA*, *accC*, transacylase *FabD*, synthase *FabF*, reductase *FabG*, *FabI*, dehydratase *FabZ*, thioesterase *FATA*, and long-chain acyl-CoA synthase *ACSL*, showed the highest enrichment among the total 22 downregulated genes. However, in the transcriptome data, we found that the stearoyl-[acyl-carrier-protein] 9-desaturase (*SAD*) gene was upregulated in wax-coated fruits. Interestingly, the octadecanoyl group during the synthesis of octadecanoic acid can be converted by the action of acyl-[acyl-carrier-protein] desaturase to octadecenoyl and then to octadecenoic acid. A heatmap of the related DEGs is shown in Figure 4A.

The extension of fatty acids is divided into two types: one occurs in the mitochondria, where two C atoms are added in each cycle of extension, the final number of C atoms is in the range of 4 < n < 16, and fatty acid degradation will be carried out after the extension is completed. The other is in the endoplasmic reticulum, where the number of C atoms is 16 or more in the cycle extension, and the final product, i.e., long-chain acyl-CoA or long-chain fatty acid will enter the wax synthesis process. Moreover, 11 genes were downregulated during endoplasmic reticulum extension, including synthase *KCS*, reductase *KCR*, dehydratase *HACD*, and thioesterase *ACOT*. Nevertheless, *KCS6*, unlike other genes, is upregulated after waxing. Furthermore, six and four differentially expressed genes were involved in fatty acid degradation and wax biosynthesis, respectively. Among the former genes, *ADH*, *ALDH*, and *ACOX* were all upregulated, and among the latter, *FAR*, and *CER* were downregulated (Figures 4B,C).

### Validation of apple fruit RNA-seq using qRT-PCR

**FIGURE 5:**
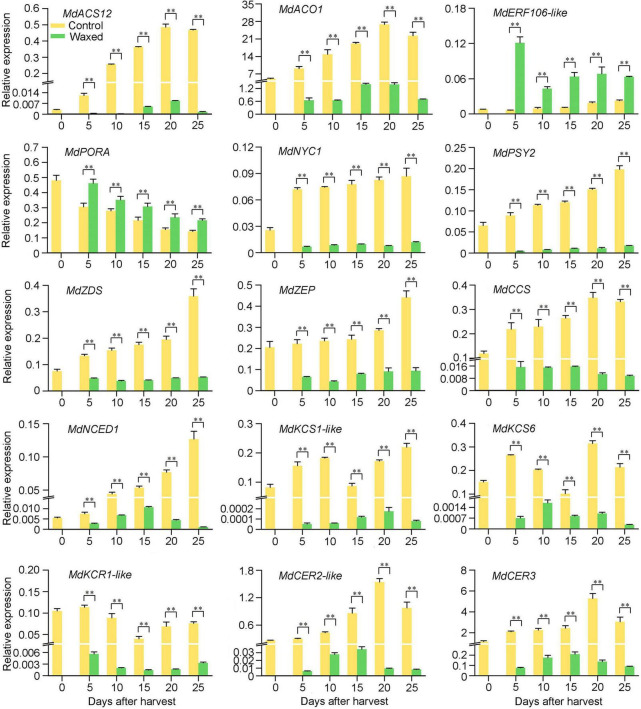
qRT-PCR validation of fifteen DEGs during apple fruit storage after wax coating treatment. Control, apple fruit not receiving treatment; Waxed, fruit treated with morpholine fatty acid salt fruit wax. The x-axis represents days of storage at room temperature after harvest. For qRT-PCR, three biological replicates were analyzed. The values represent means ± SE. Statistical significance was determined using a Student’s *t*-test, and ^**^p < 0.01.

To confirm the findings of RNA-seq, we randomly selected DEGs from the KEGG pathways as described above for qRT-PCR analysis. Fifteen DEGs were selected from different pathways such as ethylene, chlorophyll, carotenoid, and wax biosyntheses and fatty acid extension. The trend of up or downregulation of these selected genes in control and waxed apple fruits was consistent with the RNA-seq results, which confirmed the accuracy of RNA-seq (Figure 5).

### Postharvest wax coating treatment inhibits pear fruit ripening and RNA-Seq analysis

**FIGURE 6:**
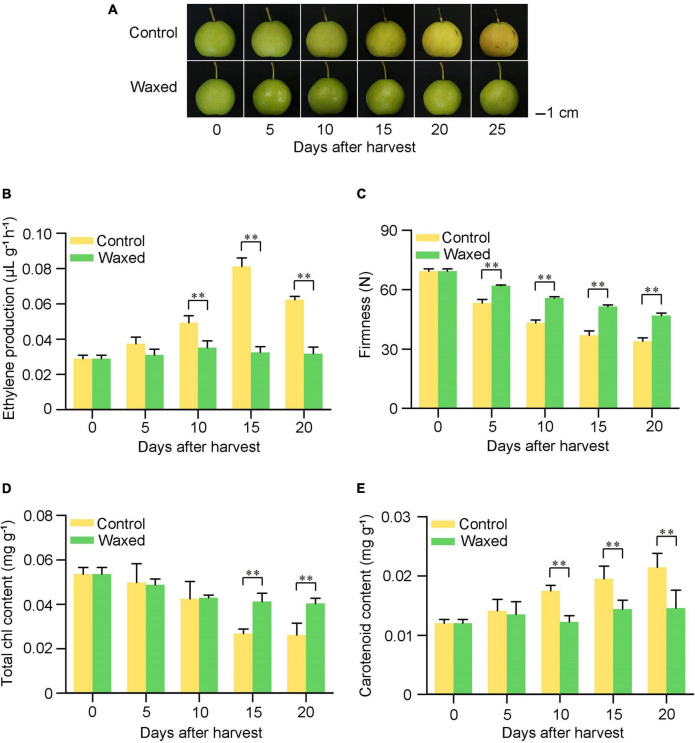
Postharvest wax coating treatment inhibits pear fruit ripening. Pear fruit were collected on the commercial harvest day (135 DAFB) in 2020 and storage at room temperature for 25 days **(A)**. After treatment, ethylene production **(B)**, fruit firmness **(C)**, total Chl content **(D)**, and carotenoid content **(E)** were measured. Control, pear fruit not receiving treatment; Waxed, fruit treated with morpholine fatty acid salt fruit wax. Scale bar = 1 cm. The x-axis represents days of storage at room temperature after harvest. Five bioligical replicates were analyzed for ethylene production, fruit firmness, and three bioligical replicates for total Chl content and carotenoid content. Values represent mean ± SE. Statistical significance was determined using a Student’s *t*-test, and ^**^p < 0.01.

To complement our research on apple fruit, we applied postharvest waxing to the pear fruit. Compared with the control fruit, the ethylene production of the wax-coated pear fruit was inhibited, and the firmness was better maintained (Figures 6A–C). Moreover, the yellowing of the epidermis of the treated fruit was inhibited, and the carotenoid and chlorophyll contents were consistent with phenotypic changes (Figures 6D,E).

Similarly, we performed RNA-seq analysis on wax-treated and -untreated pear fruit samples stored for 10 d. The two samples were named Pear-wax and Pear-con, respectively. The statistical summary are listed in Supplementary Table 2. In the transcriptome data, there were 3,247 DEGs, of which 1,349 genes were upregulated, and 1,898 genes were downregulated (Supplementary Sheet 4). We performed functional clustering and enrichment analysis for these DEGs using the KEGG database, and similar to our results obtained with the apple fruit, we identified enrichment for plant hormone synthesis and signal transduction, chlorophyll and carotenoid synthesis and degradation, and fatty acid with wax biosynthesis (Supplementary Sheet 5). Upon further analysis of the DEGs, we observed that wax coating resulted in the downregulation of genes involved in ethylene syntheses, such as *metK*, *ACS*, and *ACO*, whereas the *ERFs* did not show a clear trend. The genes associated with chlorophyll syntheses, such as *ChlH*, *MPEC*, *PORA*, and *CAO*, were upregulated. Except for *VDE*, the remaining DEGs in the carotenoid pathway were downregulated, which was consistent with our observations for the apple transcriptome. In the process of fatty acid synthesis, elongation, and degradation, related DEGs, such as *accC*, *FabD*, *FabF*, *KCS*, *ADH*, and *ALDH*, were downregulated after wax treatment. However, unlike the apple transcriptome, *FATA*, *ACSL*, and *KCR* genes were upregulated. Moreover, in the process of wax biosynthesis, the DEGs of pears were different from those of apples, which may be caused by the different effects of wax coating on wax conversion in the two fruits.

**FIGURE 7:**
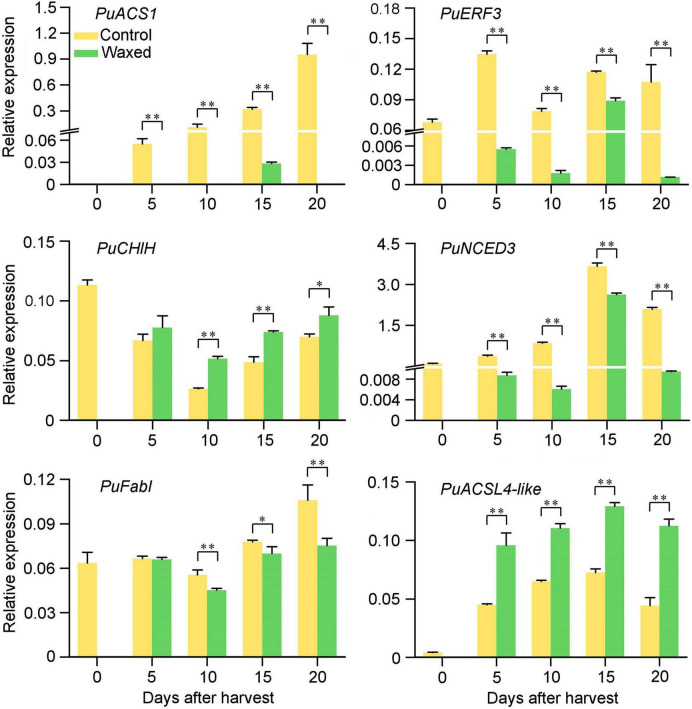
qRT-PCR validation of six DEGs during pear fruit storage after wax coating treatment. Control, apple fruit not receiving treatment; Waxed, fruit treated with morpholine fatty acid salt fruit wax. The x-axis represents days of storage at room temperature after harvest. For qRT-PCR, three biological replicates were analyzed. The values represent means ± SE. Statistical significance was determined using a Student’s *t*-test, and *p < 0.05, ^**^p < 0.01.

To confirm these observations from the RNA-seq analysis of pear fruit, we randomly selected six DEGs for qRT-PCR analysis (Figure 7). The test results show that the RNA-seq data is reliable.

In conclusion, postharvest wax treatment of pear fruit can delay fruit ripening, and the genes associated with ripening and fatty acid pathways showed a very similar expression profile to that observed for apple fruit.

## Discussion

In this study, morpholine fatty acid salt fruit wax at a concentration of 80% was used for postharvest waxing of fruits, and RNA-seq technology was used to analyze DEGs in apple and pear fruits after waxing. The KEGG database was used for functional clustering and enrichment analysis of the DEGs. The highest enrichment for DEGs was observed for plant hormones, chlorophyll, carotenoids, and fatty acid pathways.

### Effects of postharvest wax coating treatment on ripening of climacteric fruits

In previous studies, the use of edible beeswax coating on mango fruit was shown to control the ripeness of the fruit and maintain the color and hardness of the peel (Sousa et al., 2021). Moreover, fucoidan coating can also effectively prolong the shelf life of mango fruits (Xu and Wu, 2021). According to our study, the postharvest wax-coated apple and pear fruits showed a peak of ethylene release during storage, a slow rate of decrease in firmness, and the fruits did not turn yellow during the later storage period. Compared with the control group, the chlorophyll content of the treated peel was higher, and the carotenoid content was lower (Figures 1, 6). This indicates that the fruit wax treatment used in this study could inhibit the postharvest ripening of climacteric fruits.

### Postharvest wax treatment and plant hormones

There have been many studies on the relationship between plant hormones and fruit ripening, and ethylene can regulate the ripening of climacteric fruit (Pech et al., 2012). The application of 1-methylcyclopropene (1-MCP) to durian fruit delayed the surge in ethylene production and inhibited the activity of *ACO* (Amornputti et al., 2016). Our transcriptome data showed that several genes involved in ethylene synthesis, such as *ACS* and *ACO*, were downregulated in wax-coated apple and pear fruits (Figure 2E). Wax treatment not only affected ethylene-related genes but also affected gene expression of several hormones such as auxin, ABA, cytokinin, and GA (Supplementary Sheets 2, 5).

In previous studies, plant hormones have been shown to be involved in regulating fruit development and ripening (Kou et al., 2021). Auxin, the first plant hormone to be discovered, controls plant developmental processes (Zhao, 2010; Krizek, 2011). In plants, natural auxins exist mainly in the form of IAA. Auxin is an essential plant hormone for fruit development, and endogenous auxin concentrations increase before fruit ripening (El-Sharkawy et al., 2014). *Aux/IAA* family members play an important role in suppressing ARF-activated gene expression levels (Dreher et al., 2006). After wax treatment, *Aux/IAA* genes were upregulated in the apple fruit; however, in the pear fruit, their differential expression trends were not consistent (Figure 2A). This indicated that auxin-related genes play different roles in the regulation of wax application to inhibit fruit ripening.

ABA has a regulatory effect on the ripening of climacteric and non-climacteric fruits (Chen et al., 2016). Moreover the exogenous application of ABA can accelerate the ripening of climacteric fruits (Mou et al., 2016). NCED is an essential enzyme in ABA biosynthesis, and mutations in the *SlNCED* gene in tomatoes reduce ABA content and delay fruit ripening (Ji et al., 2014). In our study, the *NCED* gene was also found to be downregulated. Overexpression of *PYL9* in tomato fruits promotes early ripening. Moreover, one *PYL* gene was downregulated in our apple transcriptome data, which was consistent with the waxing inhibition of fruit ripening (Figure 2B). In summary, wax coating inhibits ABA biosynthesis, and ABA is involved in fruit ripening.

Cytokinins and GAs are important plant hormones. According to the KEGG pathway analysis, we found some DEGs in wax-coated fruit (Figures 2C,D). However, the DEGs in apple and pear fruits differed from each other; this may be due to the different effects of wax coating on hormonal signaling in the two species.

### Chlorophyll and carotenoids in postharvest waxing treatment

During the natural ripening of the fruit, there is a change in the color of the epidermis, which is mainly caused by a decrease in the chlorophyll content and a concomitant increase in the carotenoid content in the peel (Figures 1, 6). After pre-treatment of pear fruit with 1-MCP, fruit ripening was inhibited, and the expression of the chlorophyll degradation gene *PcNOL* was downregulated in the treatment group (Zhao J. et al., 2020). Similarly, 1-MCP treatment delayed the breakdown of chlorophyll in cabbage (Hu et al., 2021). In our study, the chlorophyll degradation-related genes *NYC* and *NOL* were downregulated in the apple transcriptome (Figure 3A and Supplementary Sheet 3). Therefore, inhibiting fruit ripening delays the degradation of pericarp chlorophyll content, regardless of wax coating or 1-MCP treatment. Chlorophyll is synthesized from the abundant amino acid glutamate precursor in the chloroplast, which is a very complex enzymatic reaction process (Masuda, 2008). In Brassica napus, mutations in the coding sequence of BnaA03.ChlH lead to decreased gene activity, affecting chlorophyll biosynthesis (Zhao C. et al., 2020). Oleocellosis of citrus can lead to increased chlorophyll content and up-regulated expression of related genes, such as *MPEC, PORA*, and CAO (Xie et al., 2019). The blocked chlorophyll synthesis of snap bean is related to the *POR* gene (Liu et al., 2020). In rice yellow-green leaf mutants, the content of chlorophyll was reduced, which was found by DNA sequencing due to the substitution of the *DVR* gene in the mutants (Long et al., 2022). Above studies have investigated the expression of the genes associated with chlorophyll synthesis. Our transcriptome data showed that the chlorophyll synthesis-related genes *ChlH, MPEC, PORA, DVR*, and *CAO* were upregulated in apple and pear fruits after wax treatment, suggesting a novel use of wax to keep fruits green (Figure 3A and Supplementary Sheets 3, 5).

Carotenoids play important coloring roles in a variety of horticultural crops, such as apples, peaches, and mangoes. During the inhibition of fruit ripening by wax treatment, the carotenoid content in “Golden Delicious” apple and “Nanguo pear” fruits increased slowly, resulting in fruits staying greener for a longer duration (Figures 1, 6). As per the transcriptome data, *PSY2*, *CHY*-β, *CCS*, and *NCED* genes were downregulated in apple and pear fruits. PSY2 catalyzes the first step in the carotenoid biosynthetic pathway and is a major rate-limiting enzyme (Zhou et al., 2022). Furthermore, MdPSY2 controls the carotenoid pathway in apples (Ampomah-Dwamena et al., 2015). In pepper fruits, PSY2 can compensate for the loss of PSY1, thereby biosynthesizing carotenoids (Jang et al., 2020). CHY-β converts β-carotene into zeaxanthin, and the expression of *CHY*-β is also increased in peach fruit with increased carotenoid content (Cao et al., 2017). In sweetpotato plants, *CHY*-β gene have the same effect (Park et al., 2015). CCS, one of the enzymes in carotenoid biosynthesis, is mainly expressed in pepper fruits (Arumingtyas et al., 2019). A study found that the expression of *CCS* genes was higher in peppers with high total carotenoid content, and two *CCS* genes were present in mature yellow peppers (Ha et al., 2007). Based on our enrichment results from the KEGG database, NCED can catalyze the conversion of violaxanthin into flavonoids and participate in ABA synthesis. The downregulation of the *NCED* gene after waxing may be due to the reduction of the catalyzed substrate because of the reduction in carotenoid content. Unlike the changing trends of other genes, *VDE* genes were upregulated in both apple and pear transcriptome data (Figure 3B and Supplementary Sheets 3, 5). VDE is a key enzyme in the lutein cycle and catalyzes the conversion of violaxanthin to zeaxanthin; therefore, we hypothesized that waxing treatment might promote the lutein cycle. Studies have shown that the expression of two EIN3 mature genes in papaya fruit promotes an increase in carotenoid content and the expression of *CpPDS2/4*, *CpZDS*, *CpLCY-e*, and *CpCHY-b* genes are also upregulated (Fu et al., 2017). An *AP2/ERF* transcription factor *MdAP2-34* can promote carotenoid accumulation within 150–170 DAFB (Dang et al., 2021). This indicates that ripening affects the accumulation of fruit carotenoids and the expression of associated genes. Consistent with our results, wax application inhibited fruit ripening and downregulated carotenoid-related gene expression.

### Fatty acids and wax biosynthesis in postharvest waxing treatment

Fruit epidermis wax can prevent water evaporation and microbial tissue invasion and enhance the color appearance during storage (Yeats and Rose, 2013).

Typically, wax biosynthesis originates from saturated fatty acids containing 16 or 18 carbon atoms (C16 or C18), forming ultra-long-chain fatty acids in the endoplasmic reticulum. These are subsequently reduced to primary alcohols by acyl groups, followed by carbonyl formation of aldehydes, alkanes, ketones, and other components (Lee and Suh, 2015). The most important component of epidermal wax is a fatty acid. In the transcriptome data from apple and pear, we observed DEGs that are associated with the processes such as fatty acid elongation, wax biosynthesis, fatty acid biosynthesis, and fatty acid degradation (Figure 4 and Supplementary Sheets 3, 5). During the storage of orange fruits, the wax content first increases and then decreases (Ding et al., 2018). Under cold storage conditions, ethephon treatment positively regulates epidermal wax accumulation in apples, whereas 1-MCP treatment inhibits this process (Li et al., 2017). Other researchers have reported that ethephon treatment increases the expression of *MdCER6*, *MdCER4*, and *MdWSD1*, the key genes for ultra-long-chain fatty acid synthesis in “Starkrimson apples, and 1-MCP decreases their expression (Li F. et al., 2019). Overexpression of *MdERF2*, a key factor in ethylene signaling, can upregulate the expression levels of Long-chain acyl-CoA synthetase 2 (*MdLACS2), Wax synthase1 (MdWSD1*), Eceriferum 4 (*MdCER4*), and Eceriferum 6 (*MdCER6*), which positively regulates the accumulation of total wax (Yingjie et al., 2022). Based on the previous studies, fruit ripening affects the wax content and the expression of related genes, and ethylene increases the accumulation of wax and changes the structure and shape of wax. In our study, waxing treatment inhibited fruit ripening, so *LACS*, *KCS*, *KCR*, and *CER* were all downregulated as per the apple transcriptome data (Figure 4). However, in the pear fruit, *LACS*, *KCR*, and *WSD1* were upregulated after treatment (Supplementary Sheet 5). This may be related to the wax coating material we selected, or it may be that the content and composition of wax in apple and pear fruits are different, resulting in differential gene expression after wax coating. Interestingly, we found that the differential genes involved in fatty acid degradation were upregulated in both apple and pear fruits (Supplementary Sheets 3, 5). Using transcriptomic data, we found that wax coating inhibited wax synthesis. To deeply explore the effect of wax coating on endogenous fruit wax, measurement of endogenous wax seems to be a promising strategy for future studies to obtain more convincing physiological data.

## Conclusion

In the present study, the wax coating treatment delayed the ripening of climacteric fruits, including a decrease in ethylene production, an increase in chlorophyll content, and an increase in the carotenoid content. Moreover, we analyzed the genes that were differentially expressed during this process by transcriptome sequencing of apples and pears. Based on the functional clustering of the DEGs, we identified that, in addition to ethylene, chlorophyll, and carotenoids, several other plant hormones play a role in this process and participate in the regulation of maturation. Further, we observed that fatty acid and wax synthesis-related genes were differentially affected by the wax application, inhibiting fruit ripening. In conclusion, our study provides a comprehensive overview of the gene networks that orchestrate the process of ripening of climacteric fruits.

## Data availability statement

The original contributions presented in this study are publicly available. This data can be found here: NCBI, PRJNA851428.

## Author contributions

HY, AW, and YS conceived this study. YS performed most of the experiments. TL and HL provided the plant materials. JL and JS treated the apple and pear fruit and took the sample. ZM and JQ participated in RNA extraction. HB analyzed the data. YS and HY wrote the manuscript. All authors read and approved the submitted manuscript.

## Footnotes

1
http://the-apple-genome.html

2
ftp://ftp.ncbi.nlm.nih.gov.html

3
http://geneontology.org.html

4
https://www.kegg.html

5
http://primer3.html
