# Supplementary material for: The molecular mechanism on suppression of climacteric fruit ripening with postharvest wax coating treatment via transcriptome
Source: Front Plant Sci. 2022 Aug 15;13:978013. doi: 10.3389/fpls.2022.978013 (PMC9421051; doi:10.3389/fpls.2022.978013)
Supplement: Supplementary file 1 [file Table_1.DOCX]

***Supplementary materials***

**The molecular mechanism on suppression of climacteric fruit ripening with postharvest wax coating treatment via transcriptome**

Yajing Si^1^, Tianxing Lv^2^, Hongjian Li^2^, Jiaojiao Liu^1^, Jiamao Sun^1^, Zhaohui Mu^1^, Junling Qiao^1^, Haidong Bu^3^, Yuan Hui^1,^*and Wang Aide^1,^*

Correspondence: Hui Yuan: [huiyuan@syau.edu.cn](mailto:huiyuan@syau.edu.cn); Aide Wang: [awang@syau.edu.cn](mailto:awang@syau.edu.cn)

**1 Supplementary Data**

Supplementary Sheet 1. All differentially expressed genes of apple fruit after waxing treatment in 2020 and 2021.

Supplementary Sheet 2. Co-upregulated and down-regulated differentially expressed genes in wax-coated apple fruit in 2020 and 2021.

Supplementary Sheet 3. Differentially expressed genes in KEGG pathways related to plant hormone, chlorophyll, carotenoids and fatty acids in wax-coated apple fruit.

Supplementary Sheet 4. All differentially expressed genes of pear fruit after waxing treatment in 2020.

Supplementary Sheet 5. Differentially expressed genes in KEGG pathways related to plant hormone, chlorophyll, carotenoids and fatty acids in wax-coated pear fruit.

**2** **Supplementary Figures and Tables**

**2.1 Supplementary Figures**


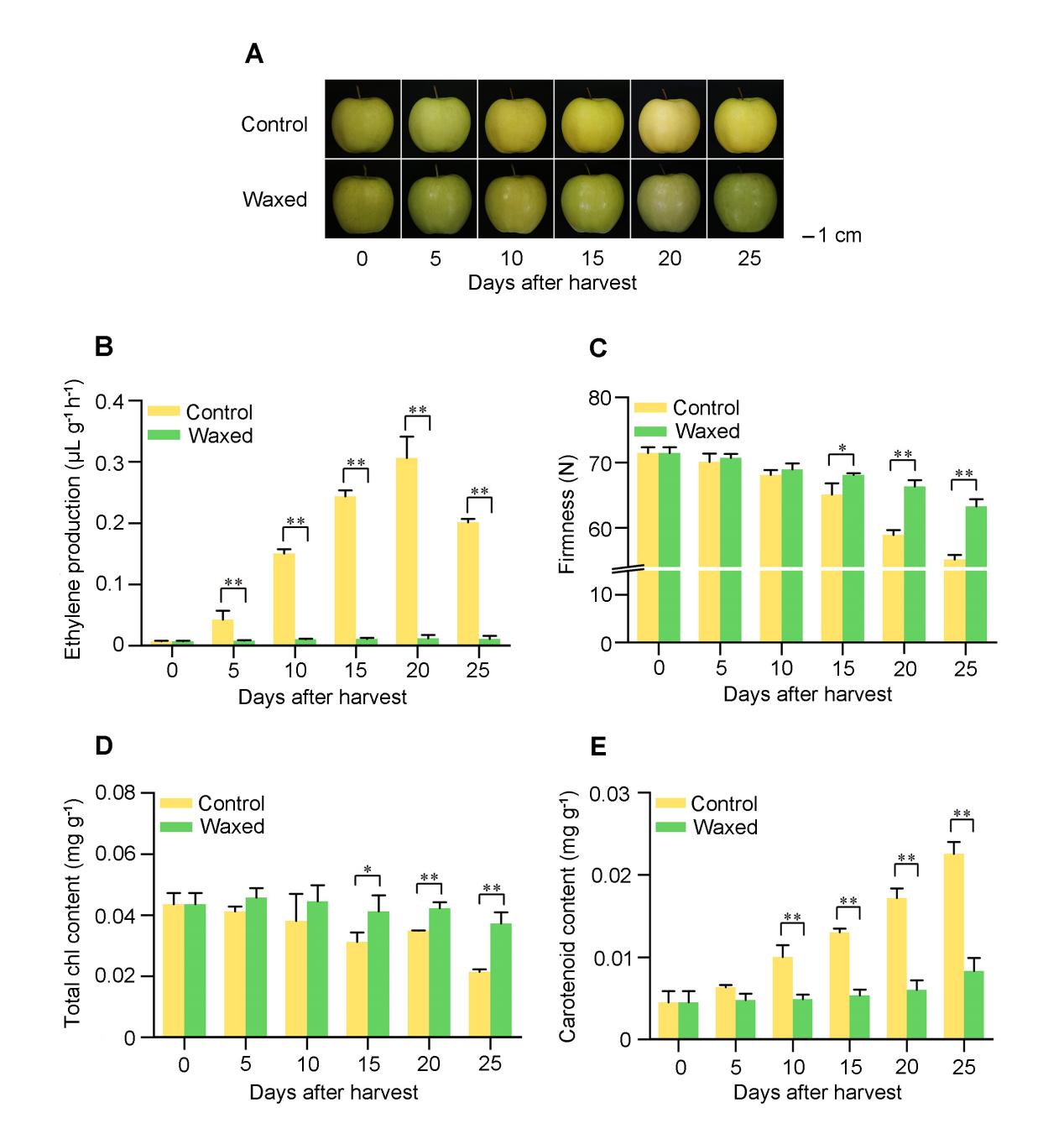


**Supplementary Figure S1.** Postharvest wax coating treatment inhibits apple fruit ripening. Apple fruit were collected on the commercial harvest day (145 DAFB) in 2021 and storage at room temperature for 25 days (A). After treatment, ethylene production (B), fruit firmness (C), total Chl content (D), and carotenoid content (E) were measured. Control, apple fruit not receiving treatment; Waxed, fruit treated with morpholine fatty acid salt fruit wax. Scale bar=1 cm. The x-axis represents days of storage at room temperature after harvest. Five bioligical replicates were analyzed for ethylene production, fruit firmness, and three bioligical replicates for total Chl conten and carotenoid content. Values represent mean ± SE. Statistical significance was determined using a Student’s t-test, and * p<0.05, ** p<0.01.


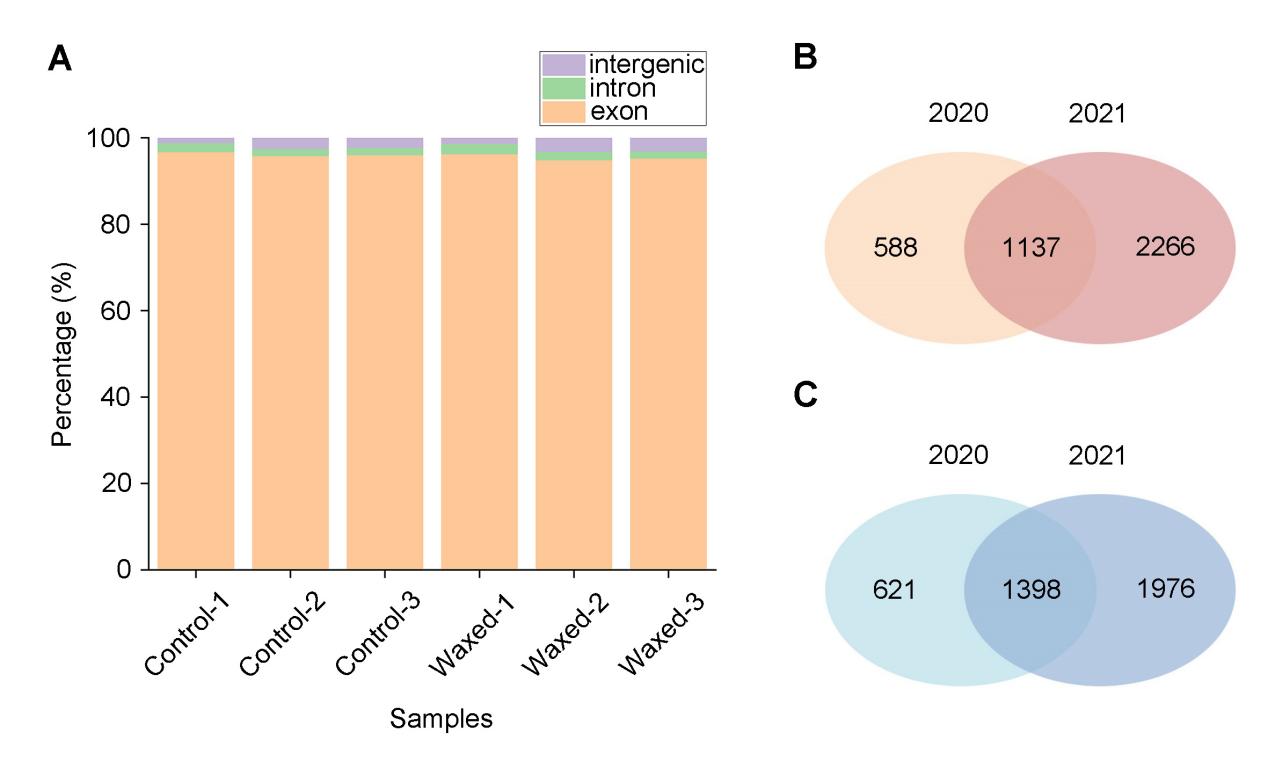


**Supplementary Figure S2.** Analysis of apple fruit RNA-Seq and DEGs in 2020-21. Categorization of the annotation region (A). Venn diagram for co-upregulation of DEGs in 2020-21 (B). Venn diagram for co-downregulation of DEGs in 2020-21 (C).


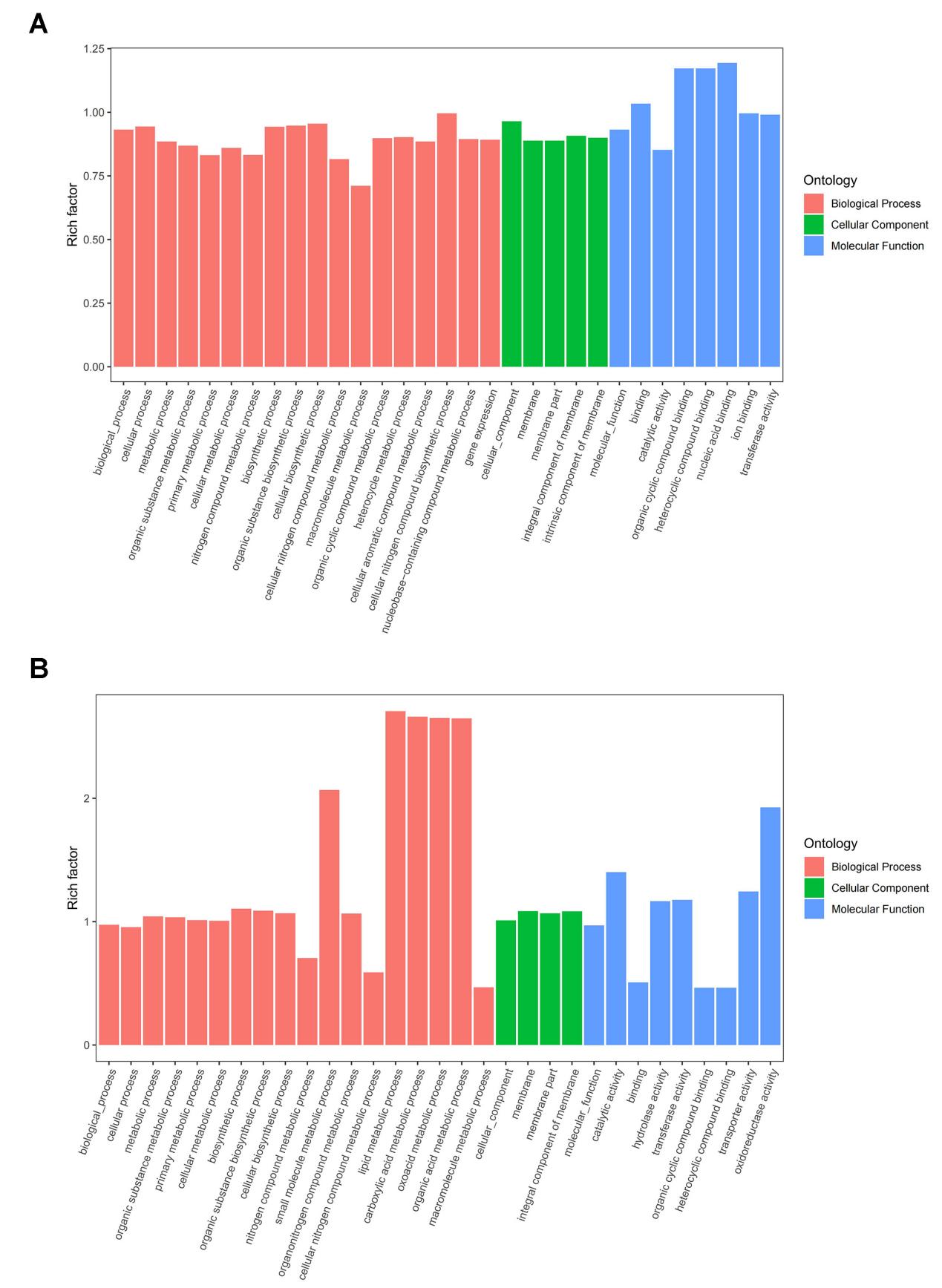


**Supplementary Figure S3.** GO classification of all commonly upregulated and downregulated DEGs in 2020-21 of apple fruit. GO classification of co-upregulated genes (A). GO classification of co-downregulated genes (B).

**2.1 Supplementary Tables**

**Supplementary Table 1. Primer sequences of DEGs for qRT-PCR.**

| **Gene id** | **Gene name** | **F-Primer** | **R-Primer** |
| --- | --- | --- | --- |
| MD01G1001600 | MdActin | TGGTGAAGGCTGGATTTG | CTGTGAGCAGAACTGGGTG |
| MD01G1186400 | probable aminotransferase ACS12 (MdACS12) | AATTGGGATTGCAGCGTACC | CCCTGTCAAAACCGGGATAA |
| MD10G1328100 | 1-aminocyclopropane-1-carboxylate oxidase 1 (MdACO1) | TGAAATTCCAAGCCAAGGAG | TTCAACTACACAAACAGTGG |
| MD07G1248700 | ethylene-responsive transcription factor ERF106-like (MdERF106-like) | CAAAATCCCGGTCCAAAAGA | TGGCAGCATCAACATCAGTG |
| MD11G1241700 | protochlorophyllide reductase (MdPORA) | ATGGCTCTTCAGGCTGCTTC | GGCTTGCTGTTGCTGTTGTC |
| MD02G1045900 | probable chlorophyll(ide) b reductase NYC1 (MdNYC1） | AGCACAAGGGGACTTGGAAA | AAGCCGCCTGCACTGTTTAT |
| MD09G1146800 | phytoene synthase 2 (MdPSY2) | GCCCTTGGAATTGCTAATCA | AGACACCCTTCTCAGCCTCA |
| MD12G1237300 | zeta-carotene desaturase (MdZDS) | ACCGAGGTCCAAAGCTGAAA | ATTTTCGTCTGCACCCACCT |
| MD02G1172400 | zeaxanthin epoxidase (MdZEP) | AATCGGAGGGTTGGTGTTTG | CCATTGATCCTGTCCCCAGT |
| MD03G1148700 | capsanthin/capsorubin synthase (MdCCS) | GAATGGTTGCGAGGCTAAGG | GCACTCTGCAATGGCTTCAG |
| MD05G1207300 | 9-cis-epoxycarotenoid dioxygenase 1 (MdNCED1) | GGCTTTGGACGTAATGGAGA | GTCTGGAATCTTTCCGGTCA |
| MD01G1087400 | 3-ketoacyl-CoA synthase 1-like (MdKCS1-like) | TGCCGTTTACCGAACAAGTG | CAGCTCGTACCACAGCGAAC |
| MD16G1043300 | 3-ketoacyl-CoA synthase 6 (MdKCS6) | TCCAATCTTGCCGGACTTCT | GGTGGCGATGAAGATGATGA |
| MD13G1107000 | very-long-chain 3-oxoacyl-CoA reductase 1-like (MdKCR1-like) | GCCTTTCTGGGTTCTTGTGC | TCCTTGAGCTTGTCCGGATT |
| MD15G1386300 | protein ECERIFERUM 2-like (MdCER2-like) | CCGAGCAATGACATGGTGTT | CCGCCTCTTCCAAATTTACG |
| MD13G1273200 | protein ECERIFERUM 3 (MdCER3) | CTGCTTGGCCTTGGGAATAC | GAAATCAACCCCCTGTTGGA |
| Pdr1g006850.1 | 1-aminocyclopropane-1-carboxylic acid synthase (PuACS1) | TTGCAACCCCAACCTAATCC | CTTTTTGCGCTTGTGGTGAG |
| Pdr8g002450.1 | ethylene-responsive transcription factor ERF003 (PuERF3) | GATCCGCCACCCTTTATTGA | CGAGGACTGAGACGCATTTG |
| Pdr12g015200.1 | magnesium-chelatase subunit ChlH (PuCHlH) | ACATTGTCGCATTGCCTTTG | TCTTCTCTGCCTTCGCCTTC |
| Pdr10g006180.1 | 9-cis-epoxycarotenoid dioxygenase 3 (PuNCED3) | ACCGGGGAGGTGAAAAAGTT | ACGTCCCATGAAACCCGTAG |
| Pdr11g024290.1 | enoyl-[acyl-carrier-protein] reductase (PuFabI) | TAAGAAGCCGTGCTGCAAAA | GAGCCTTCGGAATGTTGAGG |
| Pdr14g011600.1 | long chain acyl-CoA synthetase 4-like (PuACSL4-like) | GACCCCAAGGGAGTATTGGT | GGCGCCAGTCCAGATAAATA |

**Supplementary Table 2. Summary of RNA-Sequencing of pear fruit waxing treatment.**

| Sample | Pear-Control | Pear-Waxed |
| --- | --- | --- |
| Raw reads | 40520776 | 46521330 |
| Valid reads | 40151406 | 45991760 |
| Q20(%) | 99.99 | 99.99 |
| Q30(%) | 98.22 | 98.32 |
| GC(%) | 46 | 47 |
| Total_mapped reads(%) | 31925283(79.51%) | 36567291(79.51%) |
| Unique Mapped reads(%) | 19859020(49.46%) | 22813757(49.60%) |
| Multi Mapped reads(%) | 12066263(30.05%) | 13753534(29.90%) |

Sample：sample name；Raw date：the total number of reads of the offline data；Valid resds: the number of reads after removal of low-quality bases, sequencing adapters, and non-carrier errors;Q20:Percentage of bases with quality value ≥ 20; Q30: Percentage of bases with quality value ≥ 30; GC: The sum of base G and C accounts for the percentage of total base number; Total mapped read: the number of reads that can be aligned to the reference genome; Unique Mapped reads: the number of reads that can only be uniquely aligned to one position in the genome; Multi Mapped reads: the number of reads that can be aligned to multiple positions in the genome.
